# TGF-β is elevated in hyperuricemic individuals and mediates urate-induced hyperinflammatory phenotype in human mononuclear cells

**DOI:** 10.1186/s13075-023-03001-1

**Published:** 2023-02-27

**Authors:** Viola Klück, Georgiana Cabău, Linda Mies, Femke Bukkems, Liesbeth van Emst, René Bakker, Arjan van Caam, HINT consortium, Ioan V. Pop, Radu A. Popp, Simona Rednic, Cristina Pamfil, Marius Farcaş, Dragoş H. Marginean, Orsolya I. Gaal, Medeea O. Badii, Ioana Hotea, Loredana Peca, Andreea-Manuela Mirea, Valentin Nica, Doina Colcear, Mariana S. Pop, Ancuta Rus, Tania O. Crişan, Leo A. B. Joosten

**Affiliations:** 1 Department of Internal Medicine, Radboud UMC, Nijmegen, The Netherlands; 2 Radboud Institute for Molecular Life Sciences (RIMLS), Nijmegen, The Netherlands; 3 Department of Medical Genetics, “Iuliu Haţieganu” University of Medicine and Pharmacy, Cluj Napoca, Romania; 4 Departement of Rheumatology, Radboud UMC, Nijmegen, The Netherlands

**Keywords:** TGF-β, Hyperuricemia, Inflammation, Mononuclear leukocytes

## Abstract

**Background:**

Soluble urate leads to a pro-inflammatory phenotype in human monocytes characterized by increased production of IL-1β and downregulation of IL-1 receptor antagonist, the mechanism of which remains to be fully elucidated. Previous transcriptomic data identified differential expression of genes in the transforming growth factor (TGF)-β pathway in monocytes exposed to urate in vitro. In this study, we explore the role of TGF-β in urate-induced hyperinflammation in peripheral blood mononuclear cells (PBMCs).

**Methods:**

TGF-β mRNA in unstimulated PBMCs and protein levels in plasma were measured in individuals with normouricemia, hyperuricemia and gout. For in vitro validation, PBMCs of healthy volunteers were isolated and treated with a dose ranging concentration of urate for assessment of mRNA and pSMAD2. Urate and TGF-β priming experiments were performed with three inhibitors of TGF-β signalling: SB-505124, 5Z-7-oxozeaenol and a blocking antibody against TGF-β receptor II.

**Results:**

TGF-β mRNA levels were elevated in gout patients compared to healthy controls. TGF-β-LAP levels in serum were significantly higher in individuals with hyperuricemia compared to controls. In both cases, TGF-β correlated positively to serum urate levels. In vitro, urate exposure of PBMCs did not directly induce TGF-β but did enhance SMAD2 phosphorylation. The urate-induced pro-inflammatory phenotype of monocytes was partly reversed by blocking TGF-β.

**Conclusions:**

TGF-β is elevated in individuals with hyperuricemia and correlated to serum urate concentrations. In addition, the urate-induced pro-inflammatory phenotype in human monocytes is mediated by TGF-β signalling. Future studies are warranted to explore the intracellular pathways involved and to assess the clinical significance of urate-TGF-β relation.

**Supplementary Information:**

The online version contains supplementary material available at 10.1186/s13075-023-03001-1.

## Background

Hyperuricemia, defined as elevated serum urate levels above the saturation threshold, is the major risk factor for gout [1]. Supersaturated serum urate precipitates into monosodium urate (MSU) crystals which deposit within joints leading to recurrent inflammatory arthritis. These gout flares are initiated by interleukin (IL)-1β production by macrophages within the synovium. In these cells, stimulation of a Toll-like receptor (TLR), by free fatty acids for instance, results in the synthesis of pro-IL-1β, while MSU crystals activate the NLRP3 inflammasome leading to active caspase-1, which processes this pro-IL-1β to mature IL-1β [2]. Based on this finding, several therapies targeting IL-1β demonstrated efficacy in treating gout flares [3].

Although presenting as an intermittent flaring condition, gout is a chronic disease [4]. It affects about 2.5–3.9% of the Western population and has become more prevalent in the last decades [5]. In addition, hyperuricemia is associated with higher incidence of comorbidities such as cardiovascular disease, type 2 diabetes, metabolic syndrome, chronic kidney disease, cancer and premature ageing [6–9]. Moreover, gout patients have an increased mortality rate of 2.21 compared with the total population, and this increase is associated with high urate levels [10]. Therefore, elucidating the mechanisms responsible for this enhanced risk to develop comorbidities caused by hyperuricemia is crucial.

Crişan et al. previously demonstrated that soluble urate leads to a pro-inflammatory phenotype in primary human monocytes characterized by increased production of IL-1β, a classical pro-inflammatory cytokine, and downregulation of IL-1 receptor antagonist (IL-1Ra), the natural inhibitor of IL-1 [11–13]. Features of proinflammatory reprogramming after urate exposure persisted for up to 6 days in PBMCs in vitro and were associated with epigenetic changes [14]. Previously published transcriptomic assessment revealed several differentially enriched pathways in primary monocytes treated with urate for 20 hours, including the TGF-β signalling pathway [15]. In line with this, several genetic variants in genes encoding activin receptors and inhibins belonging to the TGF-β superfamily were found to be associated with serum urate concentrations [16]. In addition, IL-37, an anti-inflammatory cytokine with an important role in gout, also functions via an interaction with SMAD3, a major intracellular signalling effector of TGF-β, further enforcing the importance of this signalling pathway in gout [17, 18].

TGF-β is generally considered an anti-inflammatory cytokine with pro-fibrotic properties which can be secreted by most immune cells [19]. It consists of three paralogs of which paralog 1 is expressed in monocytes. When TGF-β is secreted, it is in inactive form bound to its latency-associated peptide (LAP) of which it can get separated via ROS, metalloproteinases and integrins [20]. The active form of TGF-β can subsequently bind to TGF-β receptor II (TGF-βRII). Upon binding of TGF-β, two TGFβRII receptors form a heterotetramer with TGFβRI, forming a signalling-competent complex able to induce the C-terminal phosphorylation of receptor-activated SMADs [19]. The activated SMADs form a complex with SMAD4 and translocate to the nucleus, where they regulate the expression of a large number of genes involved in e.g. fibrosis and immune signalling. Independently of SMADs, TGF-β can also signal via, e.g. TAK1, ERK and the PI3K-Akt pathway, of which the latter was also shown to be involved in urate priming [15].

In myeloid cells, the function of TGF-β depends on the specific nature of the activating conditions. Generally, TGF-β stimulates cells at the resting state, whereas activated cells are inhibited [21]: in activated monocytes, TGF-β inhibits MyD88-dependent TLR- and IL-1R signalling pathways by promoting MyD88 degradation [22]. However, TGF-β alone can induce gene expression of IL-1 in peripheral blood monocytes [23–25].

Taken together, hyperuricemia has pro-inflammatory effects in human monocytes and is a risk factor for gout and its associated comorbidities. Previous findings suggest TGF-β pathway might be a relevant target to assess in relationship to the inflammation elicited by urate. Therefore, in this study, we explore the role of TGF-β in the context of hyperuricemia and urate induced reprogramming of myeloid cells.

## Materials and methods

### Volunteers

For the discovery cohort, blood from 9 gout patients (8 male, 1 female, mean age 66.2 years old) was used for identification experiments. Blood from 7 healthy volunteers was used as a control (6 male, 1 female, mean age 60.4 years old). All volunteers gave informed consent to use leftover blood for research purposes. Blood draw from healthy volunteers were approved by the Ethical Committee of the Radboud University Medical Center (no. NL32357.091.10 and no. NL42561.091.12)

Our validation cohort consisted of 197 individuals with normouricemia, 179 individuals with hyperuricemia without gout, and 195 patients with gout. All study participants in the gout group were included if they corresponded to the ACR/EULAR 2015 classification criteria with a score of 8 or higher. Thirty-six patients presented to the rheumatologist with acute flares. For gout management, allopurinol was used either alone or in combination with NSAIDs and/or colchicine. Volunteers in the hyperuricemia group were included based on serum urate levels of 7 mg/dl or higher and negative history of gout flares. None of the individuals with hyperuricemia were treated with urate-lowering therapies. All participants were included in Cluj-Napoca, Romania, as part of the HINT project (supported by the Romanian Ministry of European Funds; P_37_762, MySMIS 103587), and both clinical characteristics and blood were collected for analysis.

For in vitro experiments, buffy coats from healthy donors were obtained after written informed consent (Sanquin blood bank, Nijmegen, the Netherlands).

### Cell isolation

Peripheral blood mononuclear cells (PBMCs) were isolated using Ficoll-gradient from whole blood of volunteers and were resuspended in RPMI 1640 supplemented with 50 μg/mL gentamycin, 2 mM L-glutamine and 1 mM pyruvate medium. Monocytes were further enriched by either adherence for 1 hour followed by washout of non-adherent lymphocytes or using Percoll gradient.

### Experimental set up

#### Ex vivo mRNA expression experiments

The PBMCs from patients and matched healthy controls were seeded on flat-bottom 96-well plates at a density of 0.5 × 10^6^ cells per well and incubated at 37 °C with 5% CO2 for an hour. Subsequently, non-adhering cells were washed away using pre-warmed PBS and the adherent monocytes were incubated with RPMI for 4 h before cells were stored in TRIzol reagent. RNA purification was performed according to manufacturer’s instructions. Subsequently, RNA concentrations were determined using NanoDrop software and cDNA was synthesized using iScript cDNA Synthesis Kit. The SYBR Green method was used to determine the mRNA expression of *TGFB1*, *TGFBR1*, *TGFBR2*, *MMP9*, *ITGAV* and *SMAD7* relative to reference gene *B2M* (primer sequences Table S1)

#### In vitro TGF-β1 signalling experiments

For mRNA expression, adherent monocytes from healthy volunteers were treated with dose-ranging concentrations of urate for 24 h. Subsequently, cells were stored in TRIzol and RNA isolation and qPCR were performed as described above.

For the pSMAD2 assessment, Percoll monocytes isolated from healthy volunteers were seeded into a 12-wells plate (1 × 10^6^ cells/well) and treated overnight with urate and, subsequently, TGF-β1 was added for the last hour. For collection of cell lysates, cells were kept on ice and lysed with lysis buffer (Cell signalling; Cat#9803) containing 1x Complete Protease Inhibitor Cocktail (PIC; Roche Diagnostics, #11697498001). Lysates were centrifuged at 25.000×g for 15 min at 4 °C, and supernatants were taken for Western blotting. Protein concentrations were determined using Pierce BCA Protein Assay Kit (ThermoFischer; Cat#23227) following manufacturer’s instructions, and equal amounts of protein were loaded in Laemmli sample buffer and separated on a 10% SDS/PAGE gel for 2 h at 120V. After running the gel, the proteins transferred to a 0.45-μM nitrocellulose membrane (GE Healthcare; Cat#10600002) using wet transfer in Towbin buffer on ice. The membrane was blocked for unspecific binding with 5% BSA-TBST followed by incubation with the primary antibody (Table S2). After overnight at 4 °C, incubation blots were washed and incubated with the secondary antibody for 30 min at RT (Table S3). After another washing step, the blots were developed using the Odyssey CLX Infrared imaging system (Licor). Quantitative assessment of band intensity was performed by Image Lab software (Bio-Rad).

#### Urate priming experiments

For urate priming experiments, adherent monocytes were primed for 24 h in RPMI supplemented with 10% human pool serum with or without urate (Sigma, 69-93-2) and recombinant TGF-β1 (R&D Systems, Catalogue number 7754-BH-005). After 24 h, cells were restimulated with 10 ng/mL ultra-pure *E. coli* LPS (InVivogen, Catalogue number tlrl-pelps). Subsequently, cell-free supernatants were collected. Secretion of cytokines was measured in supernatants using ELISA kits for IL-1β, IL-6, IL-1Ra and TGF-β (R&D Systems, Catalogue number DY201, DY206, DY280 and DY240 respectively).

To inhibit TGF-β receptor signalling, three inhibitors were used. The ALK4/5/7-kinase inhibitor SB-505124 (Sigma) in a concentration of 5 μM with DMSO as solvent control, 5Z-7-oxozeaenol (100 nM) dissolved in DMSO (Tocris Bioscience) and a blocking antibody against TGF-β receptor II (AF-241-NA, R&D systems) with mouse IgG1 as the isotype control (10 μg/mL). Cells were pre-incubated with the inhibitor for 0.5 h before adding urate.

### Proteomics

Serum samples from controls (*N* = 196), hyperuricemic (*N* = 173) and gout patients (*N* = 213) collected and stored at – 80 °C were used for commercial targeted serum proteomics analysis (Olink, Uppsala, Sweden). Olink Target 96 Inflammation panel measures 92 protein biomarkers and four internal control samples, using 1 μl serum sample, by multiplex proximity extension assays [26]. The method uses two specific DNA-labelled antibodies for each protein that upon target binding come in close proximity to each other and allow the formation of a PCR reporter sequence that is quantified by real-time PCR (qPCR). Results are generated from cycle threshold (Ct) values. The normalized protein expression (NPX) values are arbitrary (log_2_ scale) units in which 1 NPX difference equals a two-fold change in protein abundance. Data pre-processing to minimize any technical intra- and inter-assay variation is performed using internal plate controls. Quality control was performed at both sample and protein levels and samples that did not pass QC were excluded. All proteomic data was corrected for age and gender before targeted analysis. In case of LIF where the majority of data were below the lower limit of detection, we chose to use the actual data as was recommended by Olink.

### Transcriptomics

Peripheral blood mononuclear cells were isolated using whole blood from normouricemic or hyperuricemic controls and from patients with gout by density gradient centrifugation using Ficoll-Paque PLUS (Sigma Aldrich). Freshly isolated cells were kept in TRIzol reagent (Invitrogen), stored at – 80 °C and were later used for commercial RNA-Seq analysis (Beijing Genomics Institute, BGI, Beijing, China). The integrity of extracted RNA was assessed using Agilent 2100 Bio. Oligo dT magnetic beads were used to capture mRNA from total RNA. Fragmented target RNA was reverse transcribed to cDNA using random N6 primers followed by end-repair and A tailing for adaptor ligation. Purified ligation products were enriched using PCR amplification followed by denaturation and cyclization of ssDNA by splint oligos and DNA ligase generating DNA nanoballs (DNBs). Sequencing of DNBs was performed on DNBseq platform.

Raw data was generated by removing reads mapped to rRNAs. Clean reads were generated using SOAPnuke software (version:v1.5.2) by removing reads with adaptors, reads with unknown bases > 10% and low-quality reads, defined as reads with a quality score less than 15 in over 50% bases. Clean reads were mapped to human transcriptome assembly GRCh37 (hg19) using bowtie2.

Read counts were normalized using DESeq2 (Version: DESeq2_1.34.0) median of ratios method using R package (Version: R4.0.4.) and were used for downstream targeted gene expression statistical analysis.

### Statistical analysis

In ex vivo experiments, Mann-Whitney *U* test and Welch ANOVA were performed to compare means between groups. For correction of multiple comparisons, Games-Howell correction was employed for proteomic data (*n* > 50 within each group) and Tamhane T2 for transcriptomics. Spearman analyses were used for correlations. For in in vitro experiments, Wilcoxon signed rank tests were employed to compare means. Differences with adjusted *p*-value < 0.05 were considered statistically significant. All analyses were done in GraphPad Prism 5.

## Results

### TGF-β is elevated in hyperuricemic individuals and correlates positively to serum urate levels

**Fig. 1:**
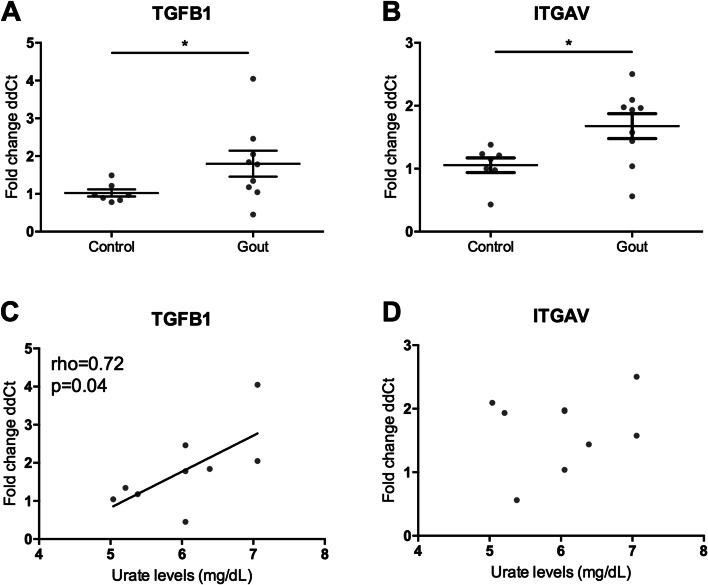
TGF-β mRNA is upregulated in gout patients and correlates to serum urate levels in the discovery cohort. PBMCs from patients with gout and matched healthy controls (HC) were isolated and adhered to a flat-bottom plate, mRNA was isolated and compared to the mean dCT of healthy controls by Mann-Whitney *U* tests *p < 0.05 (**A**, **B**). Serum urate levels were correlated to relative mRNA expression levels and analysed by Spearman’s correlation (**C**, **D**)

As a first exploration, mRNA levels of *TGFB1*, *TGFBRI*, *TGFBRII* and three TGF-β target genes *ITGAV*, *MMP9* and *SMAD7* were compared between untreated adherent monocytes of patients with gout and age and sex matched healthy controls. Expression levels of *TGFB1* were increased in individuals with gout compared to controls, and *ITGAV* mirrored this expression of *TGFB1* (Fig. 1A, B). We identified no change for *TGFBRI*, *TGFBRII*, *MMP9* and *SMAD7*. Moreover, serum urate levels correlated positively to *TGFB1* mRNA expression in patients with gout (Fig. 1C).

**Fig. 2:**
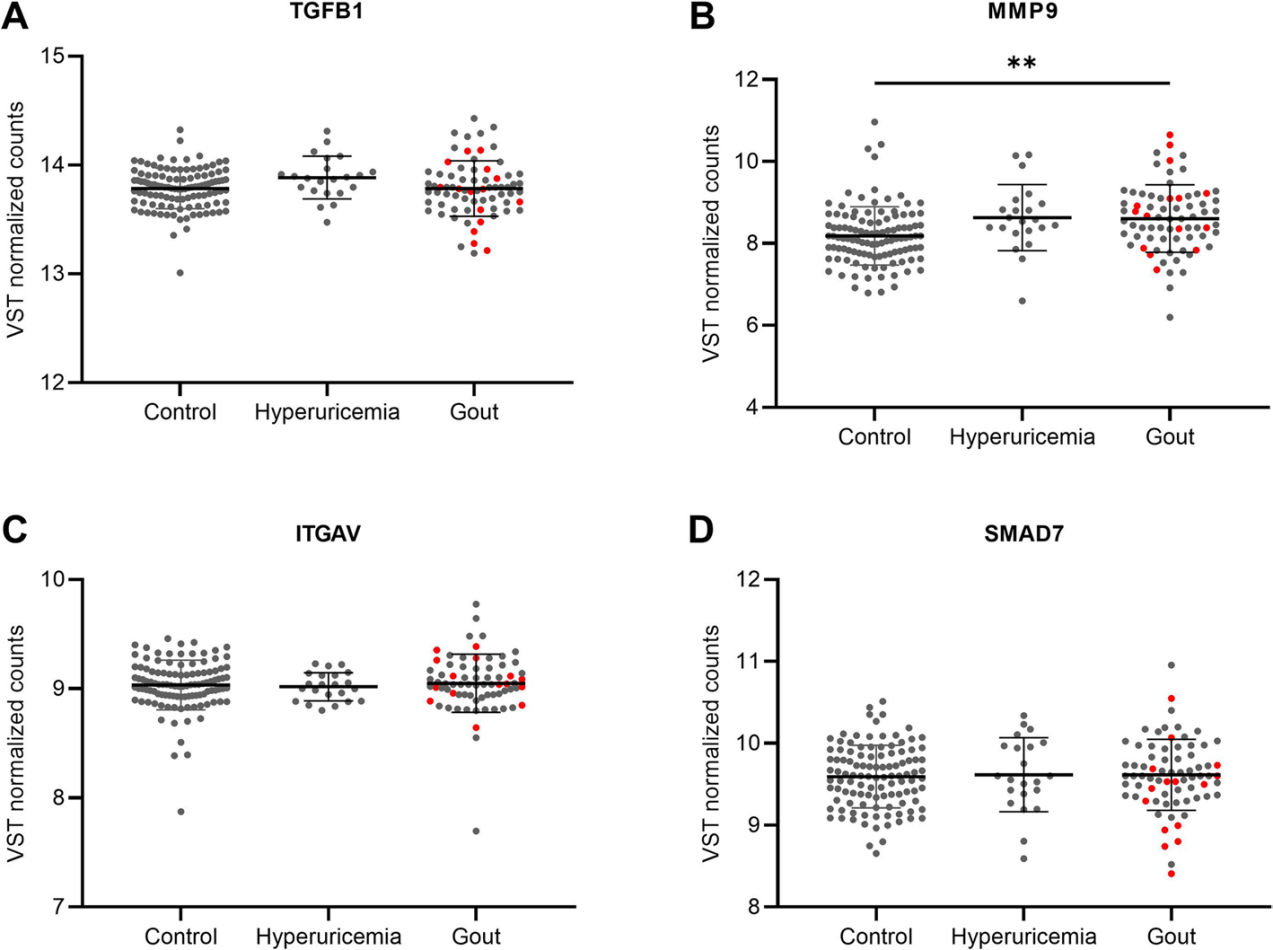
mRNA expression of TGF-β and downstream targets in PBMCs from individuals with hyperuricemia or gout. PBMCs from individuals with normoruricemia (*n* = 110), hyperuricemia (*n* = 22) and gout (*n* = 72) of which 15 flaring (marked in red) were isolated and transcriptomics were analysed. Relative mRNA expression of TGFB1 (**A**), MMP9 (**B**), ITGAV (**C**) and SMAD7 (**D**) are shown. Lines represent means with SD. Means were compared by Welch ANOVA with Tamhane’s T2 multiple comparisons test. **p < 0.01

To validate these findings in a larger cohort, mRNA expression of the same genes (*TGFB1*, *TGFBRI*, *TGFBRII*, *ITGAV*, *MMP9* and *SMAD7*) was assessed in unstimulated PBMCs from individuals with normouricemia, hyperuricemia and patients with gout (HINT study). Within the group of gout patients we observed no differences between intercritical and flaring patients. We observed no significant difference in receptor expression or *TGFB1*, *ITGAV* and *SMAD7*, but the downstream target *MMP9* was increased in patients with gout compared to controls (Fig. 2A-D).

**Fig. 3:**
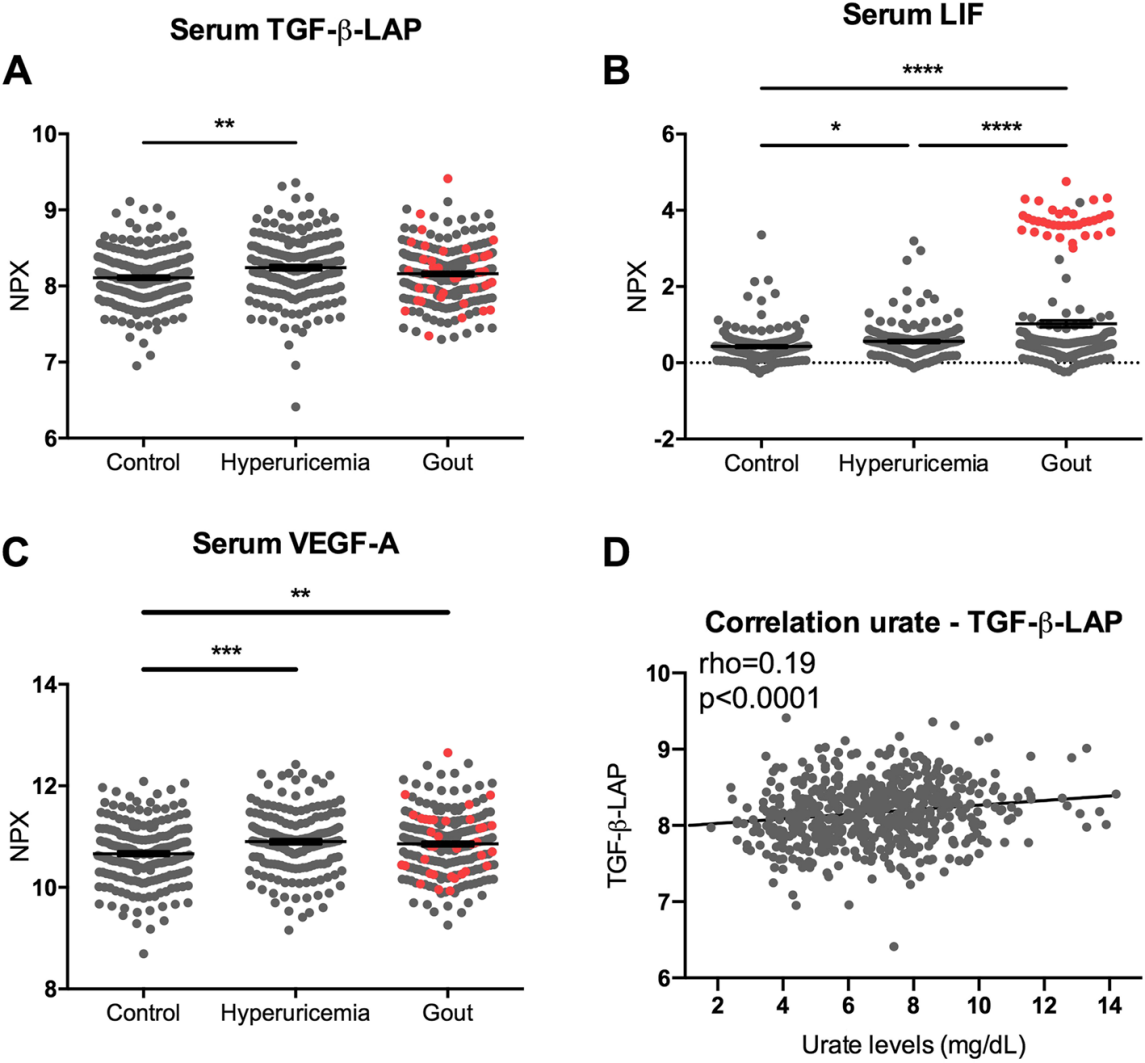
Serum TGF-β-LAP, LIF and VEGF-A levels are elevated in individuals with hyperuricemia and TGF-β-LAP correlates positively to serum urate. Serum proteins were analysed by Olink proteomics panel. Flaring gout patients (*n* = 36) are marked in red dots. NPX was shown for TGF-β-LAP (**A**), LIF (**B**) and VEGF-A (**C**). Means were compared by Welch ANOVA with Games-Howell’s multiple comparisons test. *p < 0.05, **p < 0.01, ***p < 0.001. Spearman correlation was used to analyse the correlation between serum TGF-β-LAP to urate levels (**D**). Serum LIF levels were significantly higher in flaring gout patients compared to intercritical gout patients (Welch’s *t*-test *p* < 0.0001)

For further assessment on a protein level, serum TGF-β-LAP and two downstream targets LIF [27] and VEGFA [28] levels were determined in the same individuals with normouricemia, hyperuricemia and gout. All were significantly higher in hyperuricemic individuals compared to controls (Fig. 3A–C). Serum LIF was significantly higher in gout patients during gout flare compared to intercritical gout. However, for TGF-β-LAP no differences were observed between controls and patients with gout, suggesting they are more related to high urate levels than to gout. To test for this hypothesis, TGF-β-LAP was correlated with serum urate levels. In line with the results observed in the discovery experiments, serum urate levels were positively correlated with serum TGF-β-LAP in all cohorts combined (Pearson’s correlation = 0,19; *p* < 0,0001; Fig. 3D).

### Urate induces TGF-β signalling in vitro

**Fig. 4:**
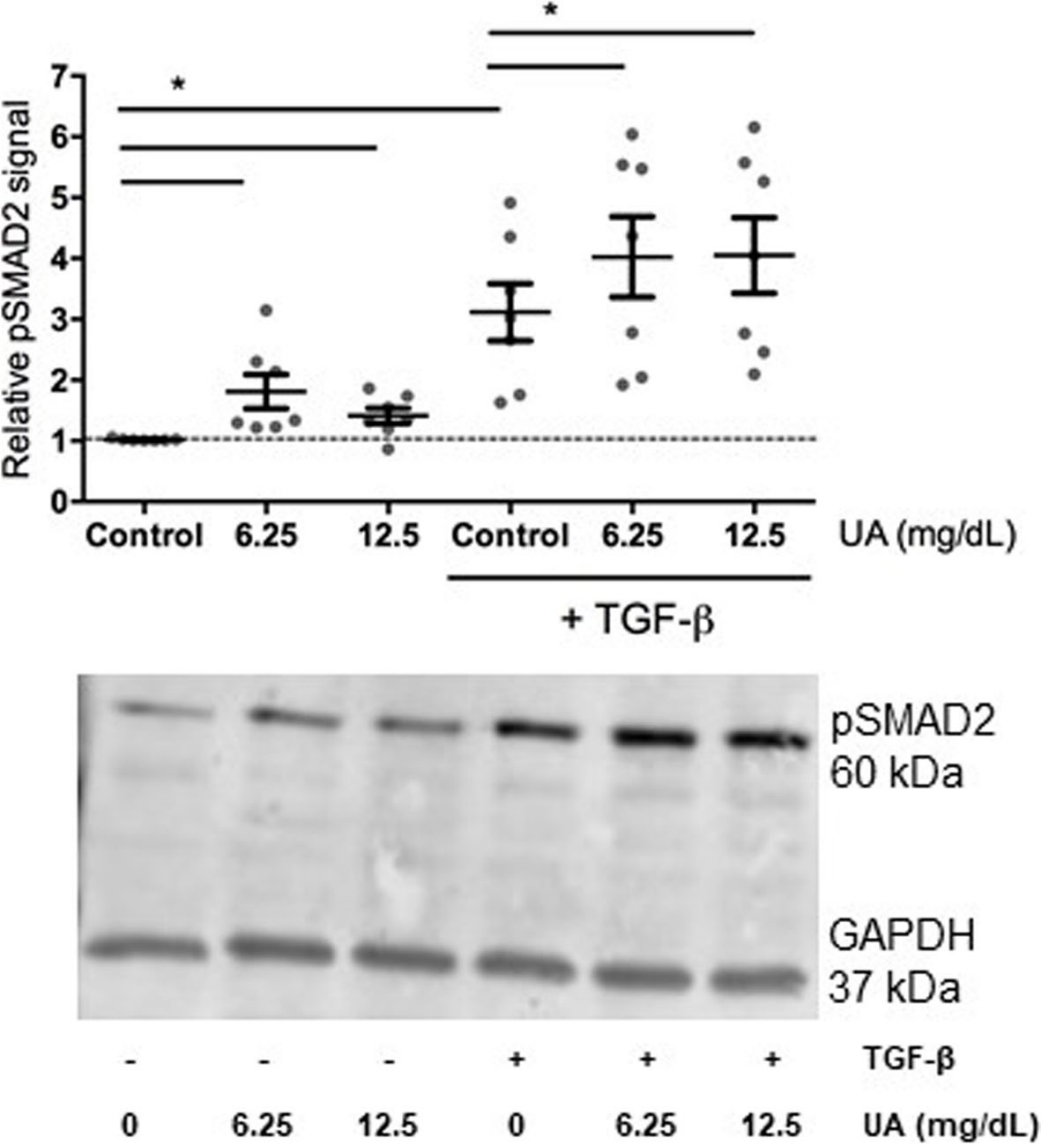
Protein expression of pSMAD2 (Ser465/467) in urate primed monocytes. The monocytes were primed overnight with no, 6.25 or 12.5 mg/dL urate followed with a stimulation of 1 ng/mL TGFβ for 1 h. Cell lysates were used for western blotting. Relative pSMAD2 expression (*n* = 7) (**A**) and a representative blot (**B**) are shown. Wilcoxon signed-rank test was used to compare means. *p < 0.05

To explore whether urate may drive TGF-β expression or production, further in vitro studies were performed. Human primary monocytes isolated from healthy volunteers treated with urate showed no elevated TGFB1 mRNA or TGF-β1 protein production after 24 h as assessed by qPCR, ELISA and luciferase bioassay (Supplemental Figures 1, 2, 3). Interestingly, in monocytes treated with urate, mRNA expression of *MMP9*, which can activate latent TGF-β to its active form, was upregulated. Moreover, the expression of *SMAD7*, a negative regulator of TGF-β signalling, was significantly downregulated compared to RPMI control condition (Supplemental Figure 1). To further assess intracellular TGF-β signalling, C-terminally phosphorylated SMAD2 was measured in urate and/or TGF-β treated monocytes. Strikingly, both urate and TGF-β induced C-terminal phosphorylation of SMAD2 showing that intracellularly TGF-β signalling was more active (Fig. 4).

### Urate induced inflammation is mediated via TGF-β

**Fig. 5:**
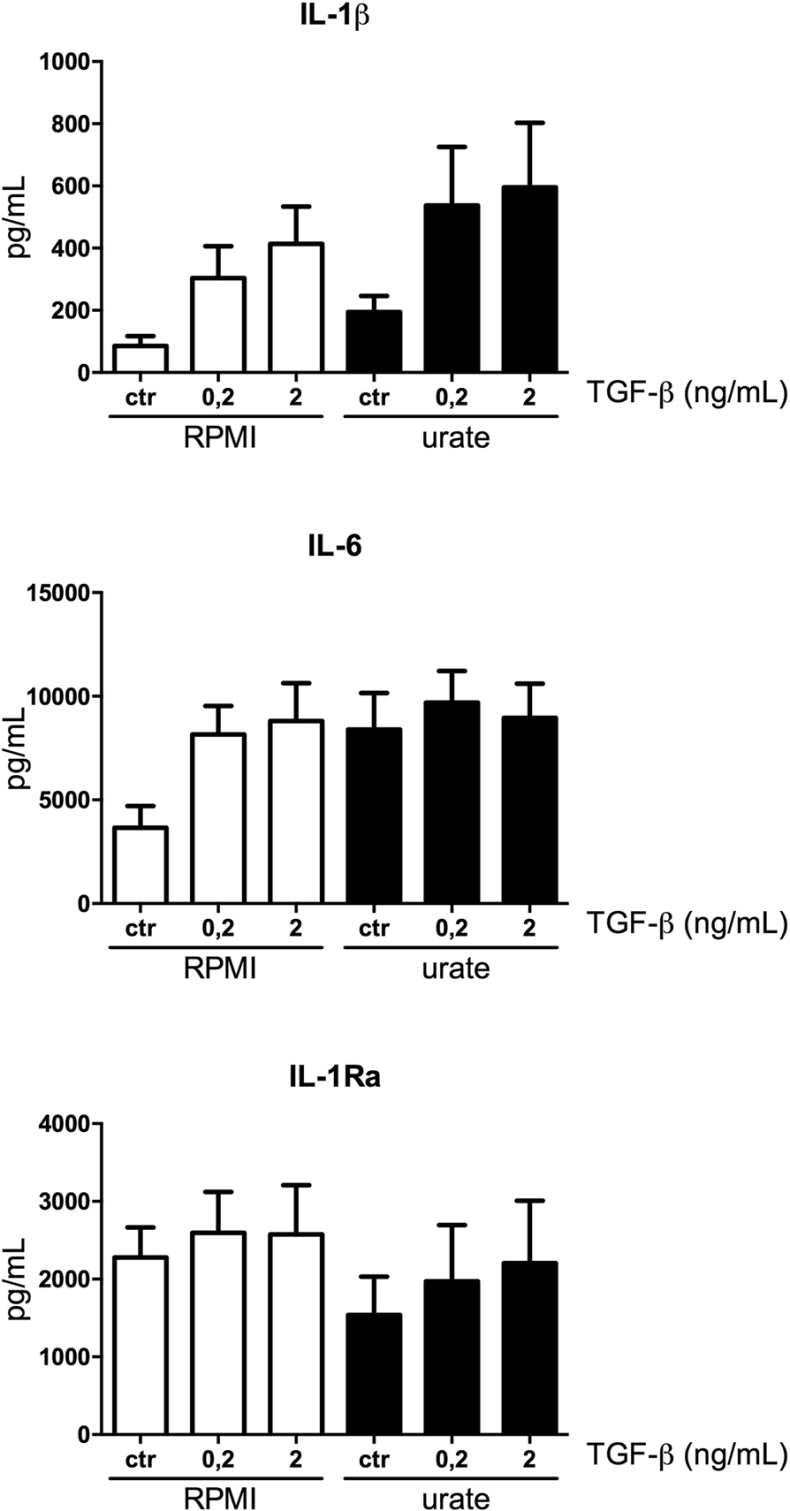
Both TGF-β and urate demonstrate pro-inflammatory effects in a priming model without a synergistic effect. Adherent monocytes isolated from healthy volunteers (*n* = 6) were treated with dose-ranging concentrations of recombinant TGF-β and/or urate (50 mg/dL) for 24 h after which cells were washed and stimulated with LPS (10 ng/mL) for 24 h. IL-1β (**A**), IL-6 (**B**) and IL-Ra (**C**) were measured in the supernatant after 48 h culture

To assess the functional consequences of enhanced TGF-β signalling, in vitro priming experiments investigating the combined effects of urate and TGF-β on cytokine production were performed. Human monocytes from healthy volunteers were treated with urate, TGF-β or a combination of the two for 24 h, washed and subsequently stimulated with LPS. Cytokine release was measured in supernatant. Both TGF-β and urate priming increased the release of IL-1β and IL-6. Whereas urate lowered IL-1Ra release, TGF-β had no effect on the production of IL-1Ra. Interestingly, we observed a small additive effect, but no synergistic effect between TGF-β and urate (Fig. 5).

**Fig. 6:**
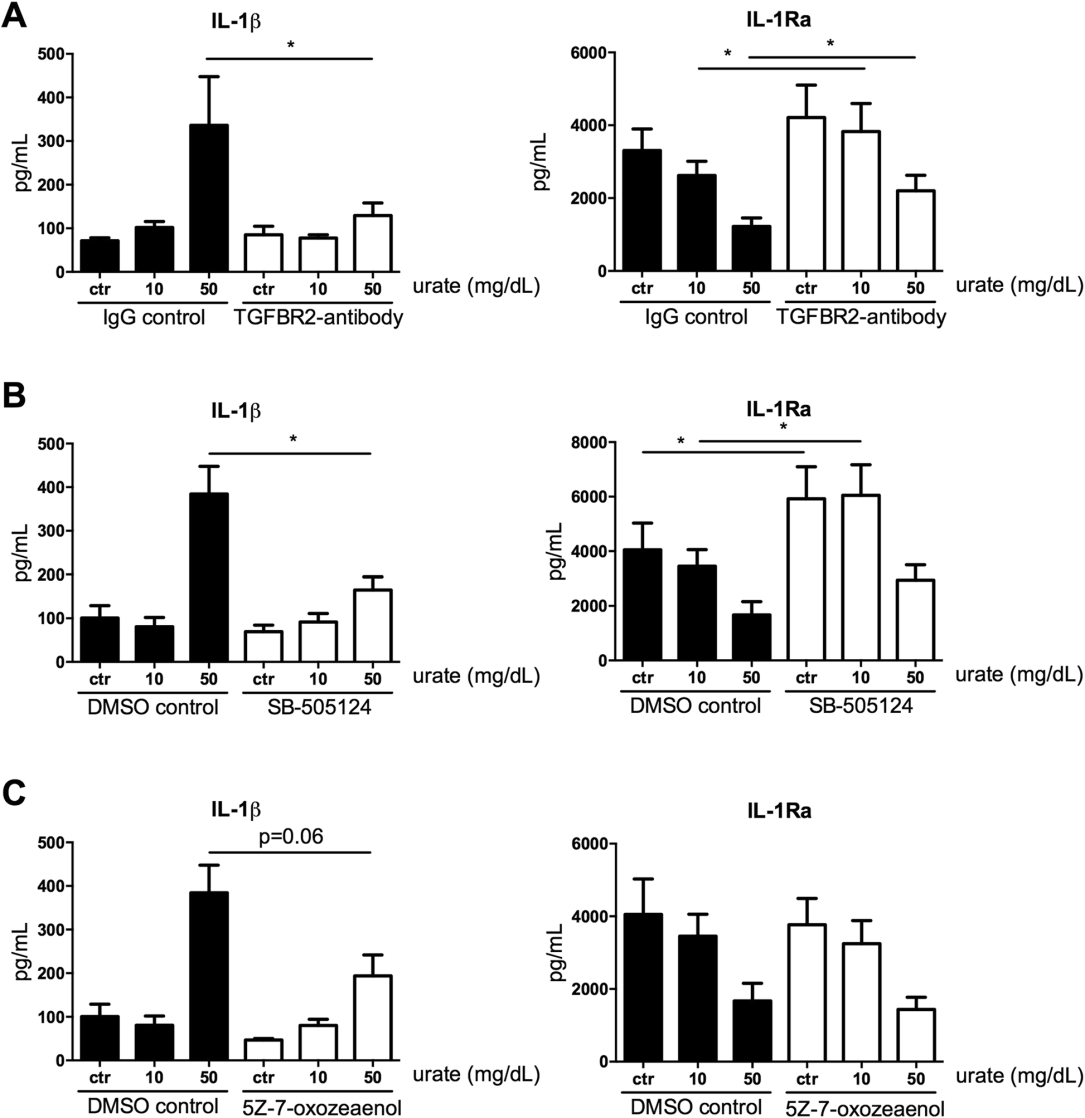
Blocking TGF-β signalling pathway partly reverses urate priming effects. Adherent monocytes isolated from healthy volunteers (**A***n* = 10; B-C *n* = 6) were treated with dose-ranging concentrations of urate (50 mg/dL) in the presence or absence of a TGF-β inhibitor for 24 h after which cells were washed and stimulated with LPS (10 ng/mL) for 24 h. IL-1β and IL-Ra were measured in the supernatant after 48 h culture. TGF-β inhibitors: a blocking antibody against TGF-β receptor II with mouse IgG1 as the isotype control (10 μg/mL), SB-505124 (5 μM) and 5Z-7-oxozeaenol (100nM) both with DMSO as solvent control. Wilcoxon signed rank test was applied to compare means. *p < 0.05

The lack of synergy between TGF-β and urate priming led us to hypothesize that the urate-induced inflammatory phenotype of the monocytes is mediated via TGF-β. Therefore, we primed human monocytes with urate in the presence of an antibody against the TGF-β receptor II. Blocking the TGF-β receptor II partly reversed the urate induced phenotype. This was shown by the fact that IL-1β production was greatly reduced and that IL-1Ra levels were partly restored (Fig. 6A). Also, SB-505124, a kinase inhibitor of TGF-β I receptors ALK 4, 5, and 7, inhibited urate induced IL-1β and restored IL-1Ra (Fig. 6B). In addition, 5Z-7-oxozeaenol, a TGF-β-activated kinase 1 inhibitor (TAK1), reduced urate-induced IL-1β, but did not affect IL-1Ra (Fig. 6C). Together, these findings pinpoint TGF-β as a potential mediator in urate-induced pro-inflammatory phenotype of human primary monocytes.

## Discussion

In this study, we assessed TGF-β in the context of hyperuricemia and gout and found that there is a role for TGF-β in urate-induced pro-inflammatory status of monocytes. In two different populations, TGF-β was elevated in subjects with hyperuricemia or gout and correlated to serum urate concentrations. In vitro, urate exposure did not directly induce TGF-β transcription or protein release in human monocytes but did induce C-terminally phosphorylated SMAD2. Moreover, urate induced elevated IL-1β production can be partly reversed by blocking TGF-β and several TGF-β receptor blockers ameliorate the urate-induced reduction of the monocyte IL-1Ra production.

In this study, we explored the expression levels of *TGFB1,* its two receptors *TGFBRI* and *TGFBRII* and three TGF-β target genes *ITGAV*, *MMP9* and *SMAD7*, the latter being a negative regulator of TGF-β signalling. In two different groups, we  observed either an increased expression of TGF-β itself in gout patients or an increase in TGF-β-LAP protein in hyperuricemic individuals. Also, the expression of the downstream targets *ITGAV* and *MMP9* were  increased in gout patients.

In our in vitro setting, we observed no change to *TGFB1* expression, a decrease in *TGFBRI* and *SMAD7* expression, while *MMP9* expression was again increased after treatment of PBMC with soluble urate for 24 h. Although both findings point towards enhanced TGF-β signalling pathway, prolonged exposure of PBMCs to elevated urate levels in vivo results to different TGF-β signalling kinetics compared to 24 h urate priming in vitro. By showing SMAD2 phosphorylation and blocking TGF-β in vitro, we were still able to demonstrate its relevance in urate-induced inflammatory phenotype.

Previously, TGF-β was studied in the context of gout. MSU crystals induce TGF-β in macrophages [29] and exogenous TGF-β inhibits MSU-crystal induced inflammation in vivo [30]. In synovial fluid, TGF-β1 is significantly elevated in acute gouty arthritis compared to osteoarthritis [31] and increases during duration of gout flare [32]. These data combined suggests an anti-inflammatory role for TGF-β in the resolution phase of gout flares. In contrast, we observed no differences in *TGFB1* expression or serum TGF-β-LAP within our gout cohort between patients during gout flare and intercritical gout patients. Possibly, TGF-β has a local effect at the site of arthritis but does not result in changes in serum protein or transcripts in circulating PBMCs. However, serum LIF protein was significantly elevated in gout patients during flare. Apart from TGF-β, LIF can also be induced by IL-1β during arthritis [33, 34], which could account for these differences. In vitro, we found that co-incubation with LPS, MSU crystals and TGF-β reduces pro-inflammatory cytokines (data not shown). However, priming monocytes with TGF-β before stimulation with LPS has pro-inflammatory effects similar to urate priming. A pro-inflammatory role for TGF-β has previously been described in adaptive immunity where TGF-β is a key regulator of T helper 17 differentiation.

Confirming our in vivo findings, a positive correlation between serum TGF-β and urate was also observed in patients with coronary artery disease [35]. This raises the question what the functional consequences are of elevated urate and TGF-β in humans. In mice, hyperuricemia induces TGF-β in renal tubular tissue [36, 37]. In parallel, allopurinol withdrawal in patients with chronic kidney disease leads to worsening of hypertension, acceleration of the rate of loss of kidney function and an increase in the urinary excretion of TGF-β [38]. The observed increase in fibrosis by urate is not limited to renal disorders. Serum urate has been described as being predictive of pulmonary arterial hypertension, a severe complication in patients with systemic sclerosis [39]. Moreover, Febuxostat, a urate-lowering drug, was shown to suppress angiotensin II-induced aortic fibrosis in mice [40].

We observed that the pro-inflammatory effects of urate were partly mediated by TGF-β. Treating monocytes with both urate and TGF-β had no obvious synergistic effect on cytokine production. Potentially, this could be explained by the presence of human serum in the culture medium which accounts for 3–4 ng/mL TGF-β already. Blocking the TGF-β pathway with several inhibitors reduced the production of IL-1β in human monocytes after stimulation with LPS. Combined with the observed SMAD phosphorylation, this suggests urate activates TGF-β signalling. One of the possible underlying mechanisms is that urate activates the TGF-β activated kinase 1 (TAK1), an important kinase in the TGF-β pathway. Uric acid molecules are capable of arresting TAK1 in an active-state conformation, resulting in sustained TAK1 kinase activation [41]. Both TGF-β induced SMAD2/3 and SMAD1/5 phosphorylation are mediated by TAK1 kinase activity. By using different inhibitors with each slightly different targets in the TGF-β signalling cascade, we could potentially pinpoint the pathways effected by urate. SB-505124, targeting ALK5, inhibits gene expression downstream of both pSMAD2/3 and pSMAD1/5, whereas (5Z)-7-Oxozeaenol, a TAK1 inhibitor, does so to a lesser extent and in a more limited number of genes [42]. In our in vitro urate priming models, SB-505124 both reduced IL-1β and restored IL-1Ra, whereas (5Z)-7-Oxozeaenol only inhibited IL-1β without affecting IL-1Ra. Possibly the difference in target gene expression accounts for this observed difference.

Another possible intracellular mechanism involved in urate and TGF-β priming is the PI3K/Akt/mTOR pathway. Although we demonstrated pSMAD2 involvement, TGF-β can also activate PI3K resulting in phosphorylation of Akt, independently of SMADs [43]. Similarly, urate priming induces pAkt in monocytes which was reversed by a PI3K inhibitor [15]. Both urate and TGF-β activate the mammalian target of rapamycin (mTOR) via PI3K/Akt pathway, thereby presumably inhibiting autophagy. Since urate induces phosphorylation of Akt within 15 min, it is uncertain whether PI3K activation is mediated via TGF-β. Further research should explore whether these shared pathways are regulated dependent or independent of each other.

Another unexplored mechanism of enhanced TGF-β signalling in urate treated monocytes is the regulation of integrin αvβ8. Human CD14^+^ monocytes activate TGF-β via the expression of the integrin αvβ8 and matrix metalloproteinase 14 [44]. Since TGF-β is always secreted as a latent complex, the function of TGF-β in the regulation of immune responses is controlled by mechanisms that regulate latent TGF-β activation.

Finally, the cellular responses to TGF-β have been shown to be altered by pro-inflammatory cytokines such as IL-1β. In chondrocytes, TGF-β induced SMAD7 could be reversed by IL-1β treatment [45]. Likewise, we observed a significant reduction in SMAD7 after urate treatment which is known to lower IL-1Ra expression. Potentially, not urate itself, but a reduction in IL-1Ra could modulate the cellular response to TGF-β similar to IL-1β. In our priming model, blocking TGF-β signalling with SB-505124 enhanced IL-1Ra release independent of urate, further suggesting a link between IL-1Ra and TGF-β signalling.

The unexplored intracellular signalling is a clear limitation of this study. Moreover, the consequence of elevated TGF-β was only evaluated in vitro. Exploring the clinical relevance of urate induced changes in TGF-β signalling is not only important for gout but also for other rheumatic diseases as osteoarthritis and systemic sclerosis. In osteoarthritis, changes in in TGF-β signalling are known to contribute to the pathogenesis [46], and serum urate was also identified as a risk factor for symptomatic knee osteoarthritis and joint space narrowing [47, 48]. Elevated serum urate is also associated with increased risk for pulmonary arterial hypertension in patients with systemic sclerosis, a complex connective tissue disease characterized by inflammation, vasculopathy and excessive fibrosis, meditated by TGF-β [39, 49].

## Conclusions

In conclusion, TGF-β is elevated in individuals with hyperuricemia correlating to serum urate levels and the urate induced pro-inflammatory phenotype in human monocytes is mediated by TGF-β signalling. Future studies are warranted to explore the intracellular pathways involved and to assess the clinical significance of the relation between serum urate and TGF-β.

## Supplementary Information

**Additional file 1: Table S1.** Primer sequences *ex vivo* experiments. **Table S2.** Primary antibodies for western blot. **Table S3.** Secondary antibodies for western blot. **Figure S1.** mRNA expression of genes in the TGF-β signalling pathway in adherent monocytes treated with urate in vitro*.* PBMCs of healthy volunteers were isolated, adhered to a flat-bottom plated and cultured in medium supplemented with 10% HPS with dose-ranging concentrations of urate. mRNA was isolated after 24h and compared to control condition by Wilcoxon matched-pairs signed rank test. *p < 0.05, **p < 0.01. **Figure S2.** Urate does not increase TGF-β release of human monocytes. PBMCs were isolated from healthy volunteers and adherent monocytes were primed for 24 hours in RPMI supplemented with 10% human pool serum with or without urate. TGF-β was measured in the supernatant by ELISA (R&D standard). **Figure S3.** Urate does not affect TGF-β bioactivity. PBMCs were isolated from healthy volunteers and adherent monocytes were primed for 24 hours in RPMI supplemented with 10% human pool serum with or without urate. Supernatant was used in a CAGA_12_-luciferase bioassay.

## Data Availability

The datasets supporting the conclusions of this article are included within the article and its additional file.

## Abbreviations

TGF: Transforming growth factor
TAK1: TGF-β-activated kinase 1 inhibitor
IL: Interleukin
IL-1Ra: Interleukin 1 receptor antagonist
PBMC: Peripheral blood mononuclear cells
mRNA: Messenger ribonucleic acid
SMAD: An acronym from the fusion of *Caenorhabditis elegans Sma* genes and the *Drosophila Mad*, mothers against decapentaplegic
MSU: Monosodium urate
TLR: Toll-like receptor
LAP: Latency-associated peptide
MMP: Matrix metallopeptidase
ITGAV: Integrin subunit alpha V
LPS: Lipopolysaccharide
PCR: Polymerase chain reaction
LIF: Leukemia inhibitory factor
VEGF: Vascular endothelial growth factor
