# Supplementary material for: TGF-β is elevated in hyperuricemic individuals and mediates urate-induced hyperinflammatory phenotype in human mononuclear cells
Source: Arthritis Res Ther. 2023 Feb 27;25:30. doi: 10.1186/s13075-023-03001-1 (PMC9969669; doi:10.1186/s13075-023-03001-1)
Supplement: Supplementary file 1 — Additional file 1: Table S1. Primer sequences ex vivo experiments. Table S2. Primary antibodies for western blot. Table S3. Secondary antibodies for western blot. Figure S1. mRNA expression of genes in the TGF-β signalling pathway in adherent monocytes treated with urate in vitro. PBMCs of healthy volunteers were isolated, adhered to a flat-bottom plated and cultured in medium supplemented with 10% HPS with dose-ranging concentrations of urate. mRNA was isolated after 24h and compared to control condition by Wilcoxon matched-pairs signed rank test. *p < 0.05, **p < 0.01. Figure S2. Urate does not increase TGF-β release of human monocytes. PBMCs were isolated from healthy volunteers and adherent monocytes were primed for 24 hours in RPMI supplemented with 10% human pool serum with or without urate. TGF-β was measured in the supernatant by ELISA (R&D standard). Figure S3. Urate does not affect TGF-β bioactivity. PBMCs were isolated from healthy volunteers and adherent monocytes were primed for 24 hours in RPMI supplemented with 10% human pool serum with or without urate. Supernatant was used in a CAGA12-luciferase bioassay. [file 13075_2023_3001_MOESM1_ESM.docx]

**SUPPLEMENTARY DATA**

**Table S1**. Primer sequences *ex vivo* experiments

| **Gene** | **Forward primer sequence** | **Reverse primer sequence** |
| --- | --- | --- |
| TGFB1 | GCCTTTCCTGCTTCTCATGG | TCCTTGCGGAAGTCAATGTAC |
| TGFBR1 | GCCAAATATCCCAAACAGATGG | ATGCCTTCCTGTTGACTGAG |
| TGFBR2 | TCCTTCAAGCAGACCGATG | TCCTTCATGCTTTCGACACAG |
| ITGAV | GGCTGCATATTTCGGATTTTCTG | CCATTCAGCTTTGTCGTCTGG |
| MMP9 | AGACCTGGGCAGATTCCAAAC | CGGCAAGTCTTCCGAGTAGT |
| SMAD7 | CATCACCTTAGCCGACTCTG | GGCCAGATAATTCGTTCCCC |
| B2M | ATGAGTATGCCTGCCGTGTG | CCAAATGCGGCATCTTCAAAC |

**Table S2**. Primary antibodies for western blot

| **Target** | **Species** | **Manufacturer** | **Product number** | **Dilution** |
| --- | --- | --- | --- | --- |
| pSMAD2 (Ser465/467) | Rabbit | Cell Signaling | 3108 | 1:1000 |
| Monoclonal anti-GAPDH | Mouse | Sigma Aldrich | SAB1403850 | 1:10.000 |

**Table S3**. Secondary antibodies for western blot

| **Secondary antibody** | **Manufacturer** | **Product number** | **Dilution** |
| --- | --- | --- | --- |
| IRDye 680RD Donkey anti-Rabbit | Licor | 926-68073 | 1:2500 |
| IRDye 800CW Donkey anti-Mouse | Licor | 926-32212 | 1:2500 |

**Figure S1 mRNA expression of genes in the TGF-β signaling pathway in adherent monocytes treated with urate *in vitro.*** PBMCs of healthy volunteers were isolated, adhered to a flat-bottom plated and cultured in medium supplemented with 10% HPS with dose-ranging concentrations of urate. mRNA was isolated after 24h and compared to control condition by Wilcoxon matched-pairs signed rank test. *p<0.05, **p<0.01


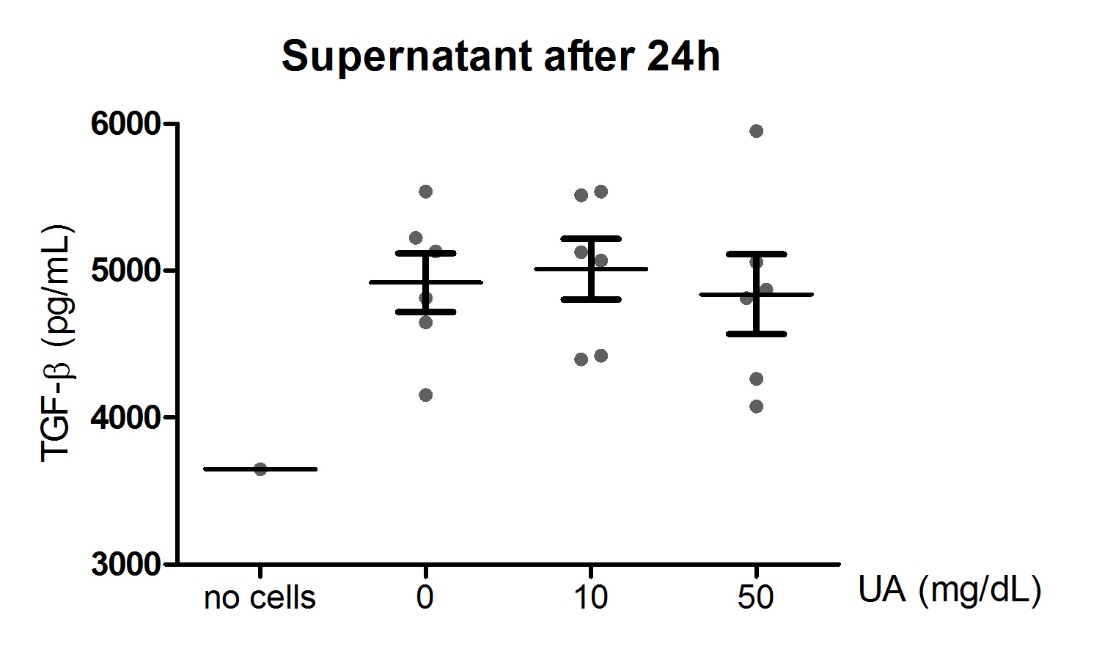


**Figure S2 Urate does not increase TGF-β release of human monocytes.** PBMCs were isolated from healthy volunteers and adherent monocytes were primed for 24 hours in RPMI supplemented with 10% human pool serum with or without urate**.** TGF-β was measured in the supernatant by ELISA (R&D standard)


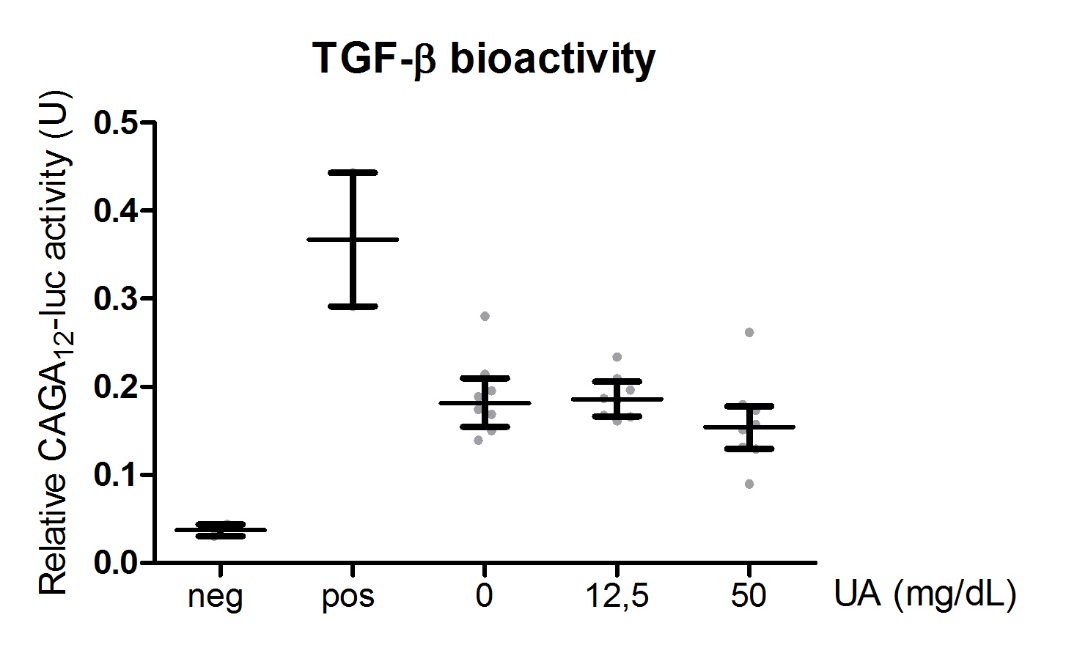


**Figure S3 Urate does not affect TGF-β bioactivity.** PBMCs were isolated from healthy volunteers and adherent monocytes were primed for 24 hours in RPMI supplemented with 10% human pool serum with or without urate**.** Supernatant was used in a CAGA_12_-luciferase bioassay.
